# Dynamic Effects of the Third Generation Bisphosphonate of Risedronate on Rat Osteoporotic Fractures for Clinical Usage Guidance

**DOI:** 10.1111/os.13158

**Published:** 2021-10-21

**Authors:** Cheng‐hui Ke, Hong‐yun Li, Dan Yang, Hao Ying, Jun Xu, Jian Wang, Hong‐wen Zhu, Lin Wang

**Affiliations:** ^1^ Department of Orthopaedics Children's Hospital of Shanghai Shanghai China; ^2^ Department of Anesthesiology Children's Hospital of Shanghai Shanghai China; ^3^ Tongji University School of Medicine Shanghai China; ^4^ Tianjin Hospital, Tianjin Academy of Integrative Medicine Tianjin China

**Keywords:** Biomechanics, Bone mineral content (BMC), Bone mineral density (BMD), Primary osteoporotic fracture (p‐OPF), Risedronate (RD)

## Abstract

**Objective:**

To better understand the risks of bisphosphonates in order to develop guidance for appropriate clinical usage, to compared femoral fracture healing at different time points and to explore the effects of Residronate on fracture healing.

**Methods:**

Osteoporosis model was achieved by ovariectomy surgery, followed by surgical incision of left femoral shaft 4 weeks after ovariectomy surgery. Three days after fracture surgery, risedronateor saline was fed by intragastric administration. X ray examination was used to check the callus formation, Bone Mineral Density (BMD), Bone Mineral Content (BMC), biomechanical, imaging and micromorphological of bone tissue as well as the trabecular bone parameters were all examined. The femoral pathology tissue of each rat was used to analyze trabecular bone parameters, including trabecular bone volume/tissue volume (Tb. BV/TV), bone surface to tissue volume ratio (BS/TV), trabecular bone mineral density (Tb. BMD), trabecular bone number (Tb. N), trabecular bone thickness (Tb. Th) and small bone Trabecular bone space (Tb. Sp).

**Results:**

*Via* X‐ray and pathologically, risedronate treatment promoted the callus forming at the fracture site during the following 6 weeks after osteoporotic fracture by X‐ray (*P* < 0.01), increased the local bone mineral density (*P* < 0.01), and accelerated the fracture healing during the first 3 weeks (*P* <0.01), but delayed facture healing in the later 3 weeks (*P* < 0.01). Risedronate increased the bone continuity of fracture at 7th week, but this phenomenon was not found at the 10th week (*P* < 0.01). Delayed fracture healing occurred locally at the fracture site. At 7th week, Risedronate may promote cartilage cells proliferating at fracture site, increase the dense of bone trabeculae and the connection of bone trabeculae, thicken the bone cortex showing better fracture healing than OPF‐Saline groups (*P* < 0.01). However, these parameter did not continue during the 7th and 10th weeks. Comparing the first and the later 3 weeks, the rats in group Osteoporotic Fracture‐Risedronate (OPF‐RD) accelerated the local fracture healing in the first 3 weeks but not in the last 3 weeks, which is consistent for the BMD and BMC among each group (*P* < 0.05). Through evaluation of bone mineral density and bone mineral content, risedronate dramatically increased the BMD at the fracture site and resulted in reduction of BMC by risedronate at the fracture site (*P* < 0.05) among each group still exist, indicating dramatic (*P* < 0.05). Through load testing, Risedronate increased the structural strength and mechanical indexes of the new callus (*P* < 0.01).

**Conclusion:**

Risedronate can improve the structural strength and mechanical index of newborn callus. Longer than 7 weeks usage of third generation bisphosphonate of risedronate does not contribute to osteoporotic fracture.

## Graphical abstract

Using a rat osteoporotic model and controls we compared femoral fracture healing at different time points to explore the effects of Residronate on fracture healing at different time points measured: (i) *via* x‐ray and pathologically; (ii) through evaluation of bone mineral density and bone mineral content; and (iii) through load testing. Our results demonstrate that Risedronate could promote fracture healing at the first 3 weeks but delayed healing at the following 3 weeks. During the first 3 weeks, risedronate could obviously increase the BMD, decrease the BMC and delay the decrease of BMC of the fracture site. During the healing process of osteoporotic fracture rats, risedronate can improve the structural strength and mechanical index of newborn callus. It strongly recommends that longer than 7 weeks usage of third generation bisphosphonate of risedronate does not contribute to osteoporotic fracture.

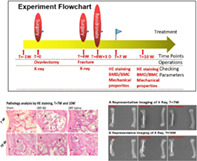

## Introduction

Osteoporosis (OP) is a chronic degenerative disease characterized by continuously low bone mass, which eventually leads to deterioration in bone strength, which is considered to be the main cause of fracture. About 10 million adults over the age of 50 in the United States suffer from OP disease^1^. OP is a high incidence bone disease in the whole body. Its characteristics are mainly the decrease of bone mineral density, the decrease of bone minerals, and the destruction of the microstructure layout of bone tissue, resulting in the decrease of bone mechanical strength and the increase of brittleness, which can easily lead to pathological fracture. Osteoporotic Fracture (OPF) is one of the most serious complications of OP, mostly in the elderly, with a high incidence. Due to the characteristics of OP, OPF is mostly comminuted and healing is slow. Because of low bone strength and high bone fragility, it is easy to cause internal fixation loosening and refracture, so the disability rate and mortality are relatively high. The World Health Organization (WHO) estimates that there are 1.66 million hip fractures worldwide each year, which is estimated to quadruple by 2050^2^. Although taking bisphosphonates (bisphosphonate drugs, BPs) has decreased the incidence of fractures in postmenopausal women, the incidence of atypical fractures has increased over the past decade^3^.

BP drug therapy is considered to be the first line of prevention and treatment for OP and has been shown to reduce the incidence of typical hip fractures in large multinational randomized controlled trials (RCTs^4^, ^5^, ^6^, ^7^). But BPs have a dual effect on bone. The first is to prevent bone loss by reducing the number of osteoclasts destroyed by preventing osteoclast formation and promoting osteoclast apoptosis, and the second is to inhibit the apoptosis of osteoblasts and osteoblasts under various pathological conditions. BPs achieve this goal by increasing bone mineral density and preventing brittle fractures^8^, ^9^. In terms of treatment, standardized BPS drug treatment can also have a positive impact on fracture healing in the short term^10^. However, long‐term use of BPs potentiates the risk of seriously inhibiting bone turnover and bone homeostasis, which may impair the ability of bone remodeling and eventually lead to the accumulation of microinjury and the decrease of bone toughness and an increase in brittleness^11^, ^12^, ^13^, ^14^. It is mainly reflected in that the incidence of OPF decreases after long‐term treatment with BPs, but the incidence of atypical fractures increases. The so‐called atypical fracture is a rare non‐invasive or minimal traumatic or stress femoral fracture, mainly subtrochanteric fracture and femoral shaft fracture^15^, ^16^, ^17^. When BPS drugs accumulate excessively in the local part of the fracture, the bone remodeling ability is obviously damaged, and the third stage of fracture healing is obviously limited, that is, the formation process of woven bone to plate bone is limited. The long‐term use of BPS drugs in the prevention and treatment of osteoporosis will damage the ability of bone remodeling and lead to the formation of new fractures, namely atypical fractures^18^, ^19^.

In this experiment, the bilateral ovaries of rats are removed for osteoporosis modeling, and then the femoral shaft fracture model is established for the first time. The purpose of this experiment is to explore: (i) the healing process of osteoporotic fractures in rats; (ii) the effect of risedronate on the healing of osteoporotic fractures in rats; and (iii) to provide guidance for the clinical use of risedronate. This study further understands and analyzes the correlation between BPS drugs and osteoporotic fractures, and provides a certain experimental basis for the long‐term clinical application of BPS drugs.

## Materials and Methods

### Experimental Groups

**Fig. 1:**
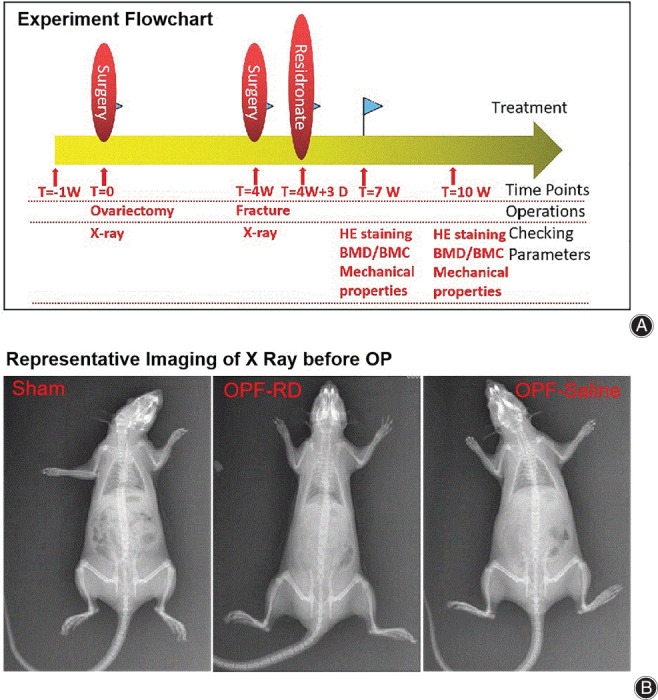
Experiment flowchart and representative X‐ray photography of rats in each group were presented. Four weeks after ovariectomy surgery, osteoporosis model was set up, at the same. Saline was administrated by perfusion. Pathological analysis by HE, BMD and BMC as well as mechanical property was carried out at the 7th week and 10th week respectively. (A) Experiment flowchart. (B) Representative imaging of X‐ray before OP. Notes: Film parameters: 41 kV, 2.80 mAs.

Sixty healthy 6‐month‐old female SD rats (clean grade) were purchased from Minhou Wu Experimental Animal Trading Co., Ltd. All rats were fed adaptively for 1 week before the experiment (Fig. 1A). All rats were weighed each week. The experimental rats were randomly divided into Sham group, OPF‐RD group and OPF‐Saline group, with 20 rats in each group. Sham group only removed the same amount of adipose tissue from the ovary. After Ovariectomy and femoral shaft fracture, OPF‐RD group was given the residronate, and the Sham and OPF‐Saline groups were given the 0.9% saline. The experimental time lasted from 6:00 to 8:00 every evening. The experiment lasted 1.5 months.

### Establishment of Osteoporosis Model

Ten per cent chloral hydrate (300 mg/kg) was injected intraperitoneally for anesthesia. The dorsal and ventral skin of rats were disinfected with alcohol iodophor. Cut the skin and separate it downward. Cut the psoas muscle under the rib 1cm away from the spine. Immediately, you can see the adipose tissue surrounding the ovary and the uterine horn closely connected with the ovary. Gently clamp the adipose tissue with curved tweezers, pull it out of the wound, ligate the fallopian tube at the upper and lower parts of the uterine horn, cut off the uterine horn and remove the ovary. Check for bleeding, push the adipose tissue back into the abdominal cavity and suture the peritoneum and muscle layer together. The Sham group only removed the same amount of adipose tissue from the ovary.

### Femoral Shaft Fracture and Administration

After the successful modeling of osteoporosis model, all rats were anesthetized with 10% chloric acid hydrate (300 mg/kg, CP Shanghai test, ≥99.0%, 250 g, Sinopharm reagent cas302‐17‐0; Shanghai). The middle part of the left femur was selected as the surgical incision site, and the skin and subcutaneous tissue were cut open, and the muscle layer was separated to expose the femur. A wire saw (Routine animal surgery equipment, Orthopedic Laboratory of the 180th Hospital of the Chinese People's Liberation Army) was in the middle of the femoral shaft and sawed through the femur. The broken end of the fracture was fixed with Kirschner (Smith & Nephew, Andover, MA, USA) wire of 1.5 mm diameter through the medullary cavity, and tissue was sutured layer by layer. Aseptic operation and intramuscular injection of penicillin 800,000 units, twice a day for 3 days after operation were guaranteed to prevent wound infection. The OPF‐RD group was given 0.1 mg/kg/daily of residronate 3 days after fracture, and Sham and OPF‐Saline groups were given the same dose of 0.9% saline. There was no death in either group, the incisions healed well, and there were no wound infections.

### Imaging Tests

All rats were anesthetized with 10% chloral hydrate (300 mg/kg, CP Shanghai test, ≥99.0%, 250 g, Sinopharm reagent cas302‐17‐0; Shanghai) to take a full‐length positive position film of the spine in supine X‐ray (Philips, Eindhoven, Germany). The bones in each rat of all groups were checked and found normal before operation (Fig. 1B). No bilateral femoral fractures and hip or knee joint lesions were found, which met the experiment requirements. Four weeks after ovariectomy, the bone mineral density of the left femur in each group of rats was measured by dual energy X‐ray absorptiometry (Hologic Corporation, Marlborough, MA, USA).

### Detection of Trabecular Bone Parameters

Rats in each group were randomly selected before modeling, 3 weeks and 6 weeks after fracture operation, and were killed by cervical dislocation. Take the pathological tissue of the left femur, remove the fixation in the pathological tissue of the femur, make the pathological tissue of the left femur into HE staining, and observe the fracture healing under the microscope. We test these trabecular parameters: bone volume fraction (BV/TV), ratio of bone surface area to tissue volume (BS/TV), bone trabecular separation (Tb. Sp), trabecular thickness (Tb. Th), Number of trabeculae (Tb. N), trabecular bone mineral density (Tb. BMD) and trabecular bone mineral content (Tb. BMC).

### Degree of Osteoporosis

Bone Mineral Density (BMD) refers to the amount of bone mineral per unit area of bone. It is an important index reflecting bone metabolism and used to analyze the changes of bone mass. The BMD values of rats in each group had differences before and after modeling, which proved that the animal model of primary osteoporosis was successfully established. Bone mineral content (BMC) measurement is to monitor whether bone tissue is normal or not, and can be used as an important index to evaluate growth, development and nutritional status. Before modeling, all rats were anesthetized by intraperitoneal injection of 10% chloral hydrate (300 mg/kg), and the BMD and BMC were measured by dual energy X‐ray absorptiometry. The femurs of rats in each group were taken at the 3rd and 6th week after fracture operation and tested again before pathology.

### Detection of Biomechanical Properties of Femur

The rats were sacrificed by cervical dislocation to make the pathological specimens of the left femur, and the 4‐point bending test was carried out with Endura TEC ELF 3200 mechanical experiment instrument and its supporting Win‐test biomechanics test software experimental instrument (Bose Corporation, Framington, MA, USA). The top span is 8 mm, the bottom span is 20 mm, and the loading speed is 1mm/min. During the measurement, the position of each specimen was kept the same, and the specimen was kept in a moist state. The maximum load was obtained by load–displacement curve, and the maximum load recovery rate of fracture was represented by the proportion of the maximum load of the fracture side to the maximum load of the contralateral non‐fracture side.

### Statistical Analysis

The data were analyzed by SPSS 17.0 software, and the measured data were expressed as (^−^ *x* ± *s*). The differences between BMD, BMC and average maximum load recovery rate were statistically analyzed. The paired *t* test was used for the comparison between BMD and BMC groups, the analysis of variance was used for intra‐group comparison, LSD test was used for variance, and Tamhane's T2 test was used for variance, *P* < 0.05 indicates statistical significance. The analysis of variance was used to compare the average maximum load and the average maximum load recovery rate between groups, and the paired *t*‐test was used for intra‐group comparison.

## Results

### Comparison of Trabecular Bone Parameters

**Fig. 2:**
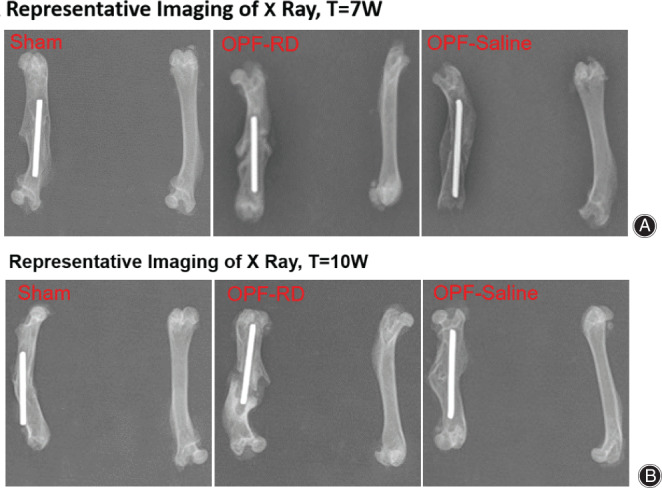
Representative X‐ray examination were performed on the fracture site of rats in each group at the end of 7 weeks and 10 weeks. (A) Representative imaging of X‐ray, T = 7 weeks. (B) Representative imaging of X‐ray, T = 10 weeks. Notes: Film parameters is 41 kV, 2.80 mAs.

In OPF‐RD group, the callus formed at the fracture site both at 7 weeks and 10 weeks, the local bone mineral density got higher and higher, the fracture healing velocity was accelerated in the first 3 weeks, and the healing was delayed during the following 3 weeks (Fig. 2A,B).

**Fig. 3:**
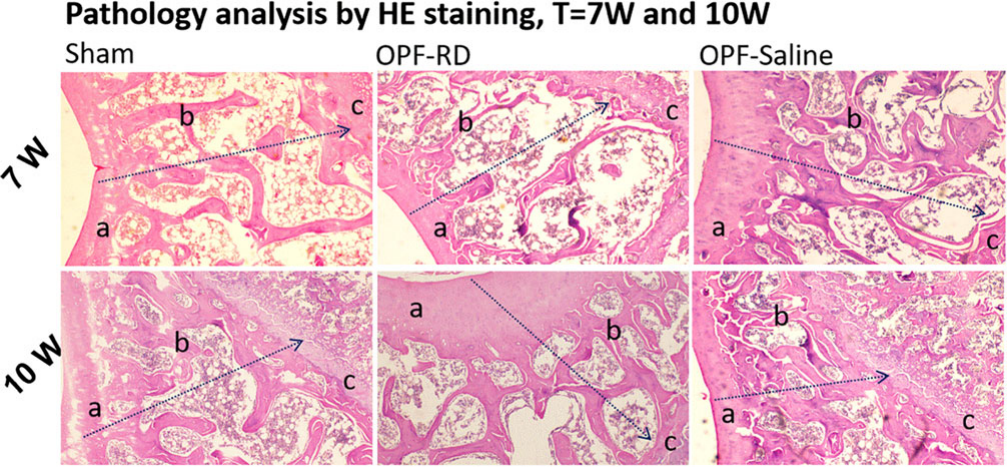
HE staining were performed on pathological tissue at the fracture site in rats of each group at the end of 7 weeks and 10 weeks. Notes: a: bone cortex; b: bone trabecula; c: for newborn cartilage cells. Magnification time: 40 × 10

In Fig. 3, A represents the bone cortex, B for the bone trabeculae, and c for the proliferation of chondrocytes. At the 7th week, pathological sectioning of rats in each group showed that more cartilage cells proliferated at the fracture site in group OPF‐RD than that in group Sham and OPF‐Saline. The data of trabecular bone parameters in each group as well as their comparison results were with the pathological results. Moreover, the bone trabeculae were denser, the bone trabeculae were better (BV/TV [1/cm], 11.64 ± 1.40, 95% *CI*: 10.99–12.30, *P* < 0.001. BS/TV [1/cm], 0.68 ± 0.06, 95% *CI*: 0.02–0.65, *P* < 0.001; Tb. BMD [mg/cm^3^], 58.42 ± 20.61, 95% *CI*: 48.77–68.06, *P* < 0.001; Tb. BMC [mg], 0.76 ± 0.14, 95% *CI*: 0.70–0.83, *P* < 0.001; Tb. N [1/cm], 0.22 ± 0.07, 95% *CI*: 0.19–0.25, *P* < 0.001; Tb. Th [cm, 0.14 ± 0.07, 95% *CI*: 0.12–0.17, *P* < 0.001; Tb. Sp [cm], −0.09 ± 0.11, 95% *CI*: −0.14 to 0.04, *P* < 0.001) connected to each other, and the bone cortex was thicker in group OPF‐RD than the corresponding parameters in the Sham and OPF‐Saline groups, which demonstrated better fracture healing. However, at the end of 10th week, fewer (BV/TV [1/cm], 20.15 ± 0.56, 95% *CI*: 19.88–20.41, *P* < 0.001; BS/TV [1/cm], 0.85 ± 0.06, 95% *CI*: 0.82–0.87, *P* < 0.001; Tb. BMD [mg/cm^3^], 129.97 ± 21.57, 95% *CI*: 119.87–140.07, *P* < 0.001; Tb. BMC [mg], 0.79 ± 0.10, 95% *CI*: 0.74–0.84, *P* < 0.001; Tb. N [1/cm], 0.68 ± 0.14, 95% *CI*: 0.62–0.74, *P* < 0.001; Tb. Th [cm], 0.14 ± 0.06, 95% *CI*: 0.11–0.17, *P* < 0.001; Tb. Sp [cm], −0.17 ± 0.09, 95% *CI*: −0.21 to 0.13, *P* < 0.001) chondrocytes, sparse bone trabeculae, poor connectivity between bone trabeculae, no obvious (*P* > 0.05) variance of bone cortex thickness and delayed fracture healing appeared in the OPF‐RD group, while no similar phenomenon showed up in the Sham and OPF‐Saline groups, when comparing the two periods, the local fracture recovered with the below parameters: (BV/TV [1/cm], −4.43 ± 1.43, 95% *CI*: −5.10 to 3.76, *P* < 0.001; BS/TV [1/cm], −0.08 ± 0.09, 95% *CI*: −0.12 to 0.36, *P* < 0.001; Tb. BMD [mg/cm^3^], −27.78 ± 20.85, 95% *CI*: −37.54 to 18.02, *P* < 0.001; Tb. BMC [mg], 0.48 ± 0.11, 95% CI: 0.43–0.53, *P* < 0.001; Tb. N [1/cm], −0.28 ± 0.08, 95% *CI*: −0.32 to 0.24, *P* < 0.001; Tb. Th [cm], −0.02 ± 0.03, 95% *CI*: −0.03 to 0.01, *P* = 0.06 > 0.05; Tb. Sp [cm], 0.03 ± 0.04, 95% *CI*: 0.01–0.05, *P* = 0.0017) healing outcome in group OPF‐RD at the end of the first 3 weeks, but delayed the healing in the following 3 weeks.

**Fig. 4:**
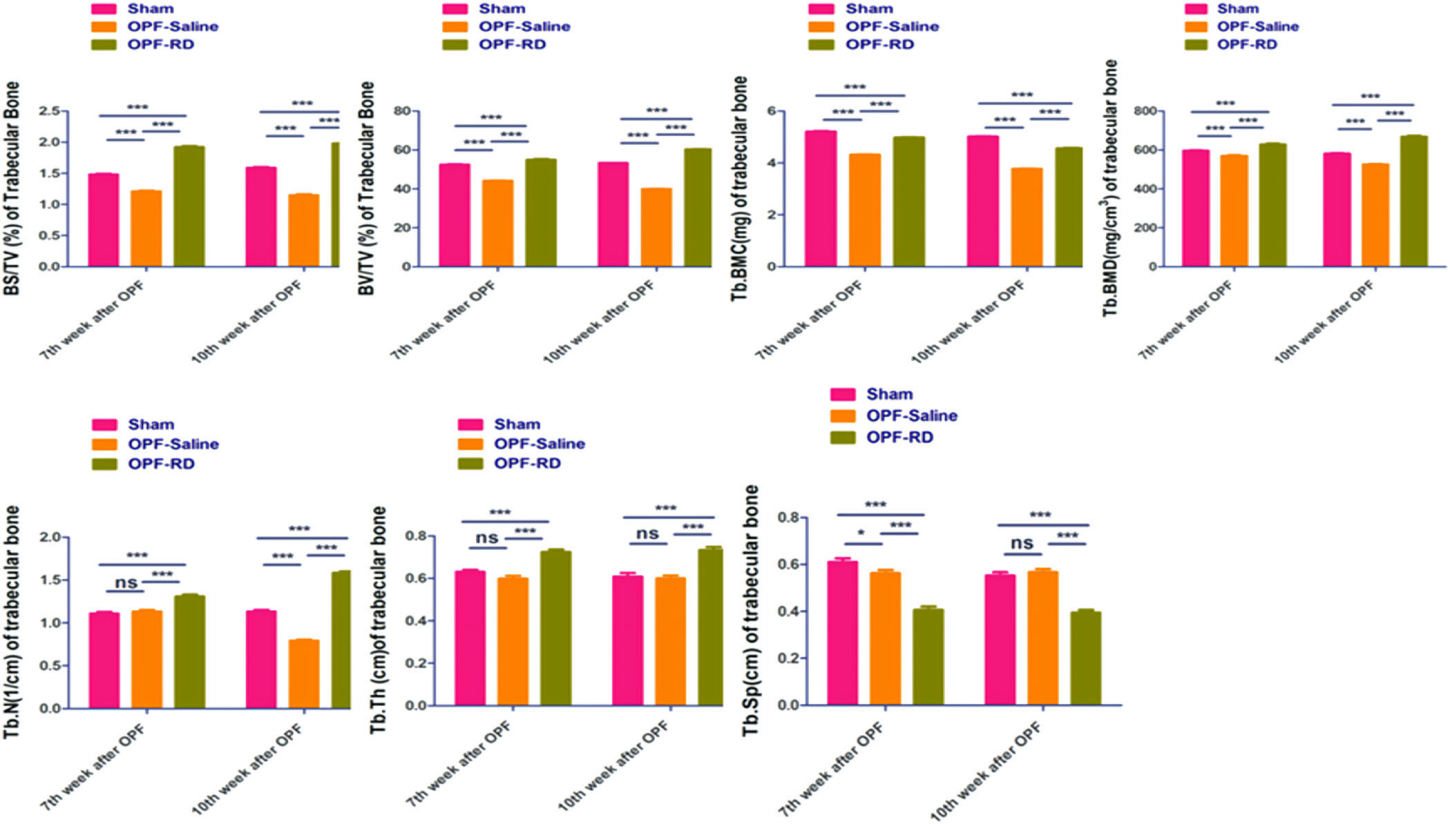
The trabecular bone parameters in various groups of rats were compared and analyzed. Note: (i) At the 7th week after OPF, the OPF‐RD group sf Tb.N was compared with the sham group, *P* > 0.05; (ii) at 7th week and 10th week after OPF, the OPF‐RD group sf Tb. Th was compared with the sham group, *P* > 0.05; and (iii) at the 10th week after OPF, the OPF‐RD group sf Tb.Sp was compared with the sham group, *P* > 0.05.

Risedronate increased the bone continuity of fracture at 7th week, but this phenomenon was not found at 10th week. Delayed fracture healing occurred locally at the fracture site. At 7th week, Risedronate may promote cartilage cells proliferating at fracture site, increase the dense of bone trabeculae and the connection of bone trabeculae, thicken the bone cortex showing better fracture healing than OPF‐Saline groups. However, these parameters did not continue during the 7th and 10th weeks. Comparing the first and the later 3 weeks, the rats in group OPF‐RD accelerated the local fracture healing in the first 3 weeks than the last 3 weeks, which is consistent for the BMD and BMC among each group (Fig. 4).

### Comparison of BMD, BMC Values

**Fig. 5:**
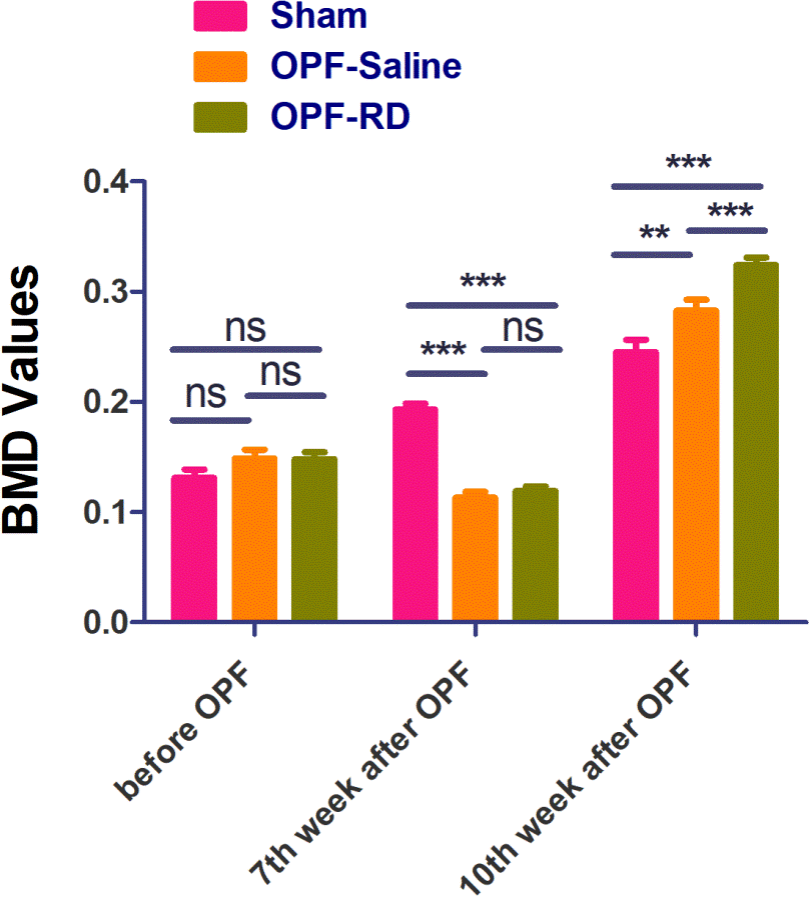
The BMD values of each group was compared and analyzed at the 7th and 10th weeks before and after OPF modeling. (*n* = 10, $\bar{x}$ ± *s*). Note: *means that *P* value is less than 0.05, but greater than 0.01. **means that *P* value is less than 0.01, but greater than 0.001. ***means that *P* value is less than 0.001. OPF‐Saline and OPF‐RD in 7th week compared with before modeling, *P* < 0.001; OPF‐Saline and OPF‐RD in 10th week compared with 7th week, *P* < 0.001.

**Fig. 6:**
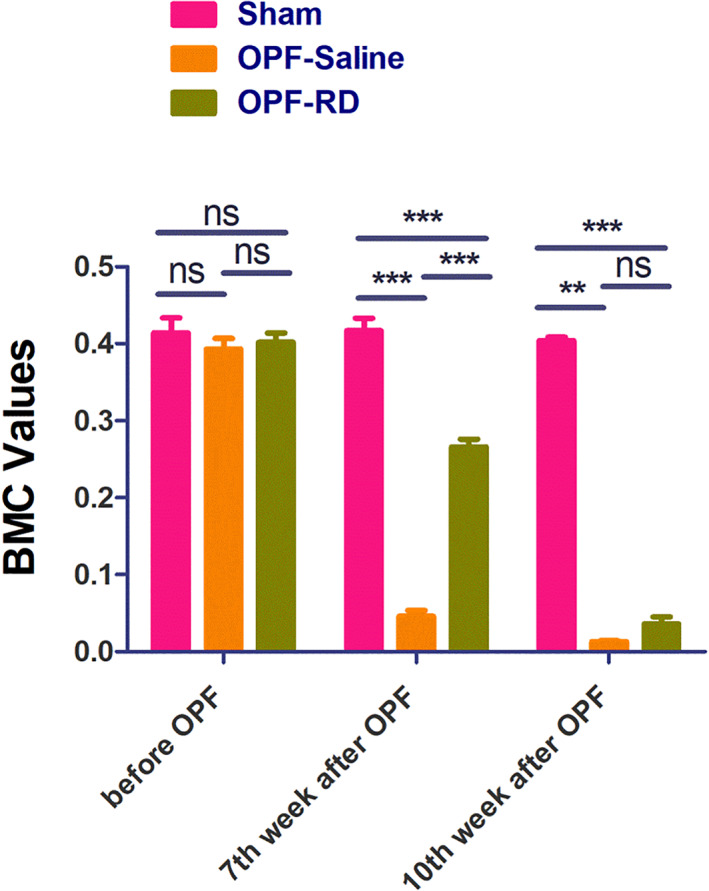
The BMD values of each group was compared and analyzed before and after OPF modeling (*n* = 10, $\bar{x}$ ± *s*). Note: OPF‐RD and OPF‐Saline group was compared at the 7th week (*P* < 0.05), and at 10th week, group OPF‐RD was compared with OPF‐Saline compared with sham (*P* < 0.05).

There was no difference (*P* > 0.05) in the mean value of BMD among groups before Osteoporotic Fracture modeling (Fig. 5). But at the end of the 7th week, apparent (sham group: 0.19 ± 0.03, 95% *CI*: 0.17–0.20, *P* < 0.001; OPF‐RD group: 0.11 ± 0.03, 95% *CI*: 0.09–0.12, *P* < 0.001; OPF‐Saline group: 0.11 ± 0.02, 95% *CI*: 0.01–0.11, *P* < 0.001) difference in the mean value of BMD among groups appeared. At the end of the 10th week, the mean value of BMD in group OPF‐RD was higher (0.04 ± 0.09, 95% *CI*: 0.01–0.08, *P* = 0.047) than that in Sham and OPF‐Saline groups, indicating that risedronate could dramatically increase the BMD at the fracture site. There was no (*P* > 0.05) difference for BMC among groups before OPF modeling, but at the end of the 7th week, obvious (sham group: 0.39 ± 0.05, 95% *CI*: 0.36–0.41, *P* < 0.001; OPF‐RD group: 0.28 ± 0.04, 95% *CI*: 0.26–0.29, *P* < 0.001; OPF‐Saline group: 0.05 ± 0.02, 95% *CI*: 0.04–0.06, *P* < 0.001) differences of BMC among each group was found. At the end of the 10th week, differences (OPF‐RD group: 0.04 ± 0.03, 95% *CI*: 0.02–0.05, P = 0.0471; OPF‐Saline group: 0.02 ± 0.01, 95% *CI*: 0.02–0.03, *P* = 0.0258) among each group still exist, indicating dramatic (0.01 ± 0.03, 95% *CI*: 0.01–0.03, *P* = 0.036) reduction of BMC by risedronate at the fracture site (Fig. 6). Through evaluation of bone mineral density and bone mineral content, it was found that risedronate could dramatically increase the BMD and reduce BMC at the fracture site.

### Comparison of Biomechanical Properties

**Fig. 7:**
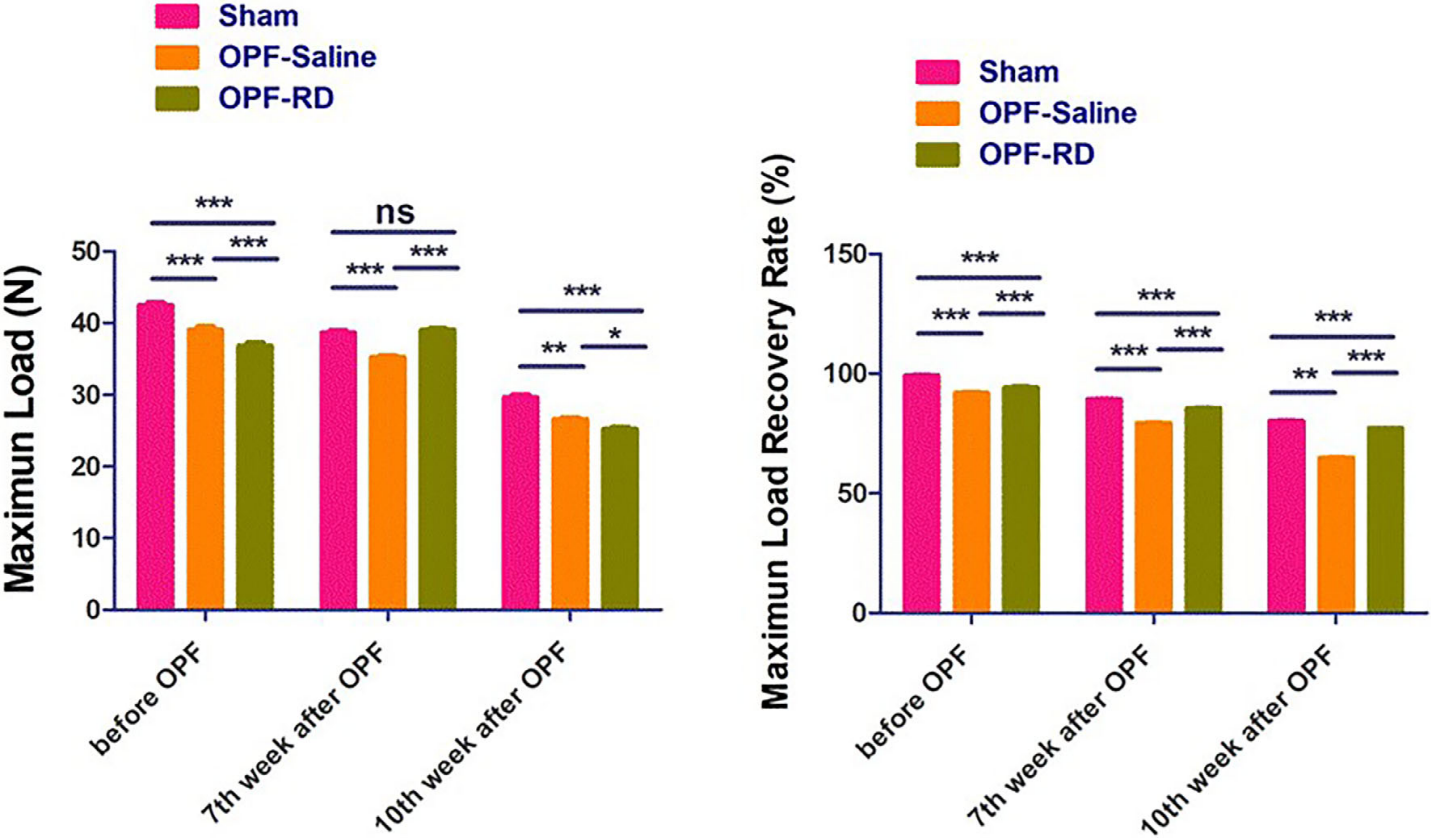
The biomechanical properties between groups and within groups were analyzed at various time points. (*n* = 10, $\bar{x}$ ± *s*). Note: (i) At every time, the comparison of components of the Maximum Load Recovery Rate between the three experimental groups is different. (ii) At 7th week, no difference of maximum load existed between group OPF‐RD and sham. (iii) In the rest of the time, the comparison of components of the Maximum Load between the three experimental groups is different.

The biomechanical properties of primary osteoporotic fracture in each group before and after modeling. No significant difference was found for the maximum load recovery rate between group OPF‐RD and group OPF‐Saline. Through load testing, Risedronate was found to increase the structural strength and mechanical indexes of the new callus maximum load (N) at the 7th week (3.88 ± 0.62, 95% *CI*: 3.59–4.17, *P* < 0.001) and 10th weeks after OPF modeling (−1.07 ± 1.17, 95% *CI*: −1.62 to 0.52, *P* < 0.001). Meanwhile, the maximum load recovery rate (%) improved at the 7th and 10th weeks after OPF modeling compared with sham group (6.40 ± 0.89, 95% *CI*: 5.98–6.81, *P* < 0.001); 7th week after OPF modeling (64.76 ± 0.55, 95% *CI*: 64.50–65.01, *P* < 0.001); 10th week after OPF modeling (12.01 ± 0.65, 95% *CI*: 11.70–12.31, *P* < 0.001; Fig. 7).

## Discussion

### General Information

Osteoporosis is a bone degenerative disease with increasing high incidence, characterized by the decrease of bone mineral density, the destruction of the microstructure of bone tissue, the decrease of bone mechanical strength and increase of brittleness, which easily leads to pathological fracture. Osteoporotic Fracture is one of the most serious complications of OP. High morbidity and mortality have always been suffered by osteoporotic fracture patients^20^. In modern medical research, prevention and control of osteoporotic fracture has become an area of focus in China. Bisphosphonate drugs, BPs, which have been used to prevent and treat osteoporotic fractures, show promise in clinical use due to their special pharmacological effect on bone. Over the past 20 years, it has been recognized that BPs have a dual effect on bones. The first is to prevent bone loss by reducing the number of osteoclasts through preventing osteoclast formation and promoting osteoclast apoptosis^21^, ^22^. The second is to inhibit apoptosis of osteoblasts in various pathological conditions^23^, ^24^. In the prevention of osteoporotic fractures, Song *et al*.^25^, reported that BPs drugs can reduce the incidence of spinal fracture by 40% to 70% and hip fracture by 40% to 50% in the current clinical application^26^. Evidence also showed that standardized BP drug therapy can have a positive impact on fracture healing in the short term^27^. However, there is a potential risk in the long‐term use of BP drugs in treating osteoporotic fractures due to the fact that long‐term use of BP drugs decreases the osteoporotic fracture incidence, but meanwhile increases the incidence of atypical fractures^18^, ^28^, ^29^, ^30^. In the results of this experiment, *via* X‐ray and pathologically, our data indicate that risedronate treatment promoted the callus forming at the fracture site during the following 6 weeks after osteoporotic fracture by X‐ray, increased the local bone mineral density, and accelerated the fracture healing during the first 3 weeks, but delayed facture healing in the later 3 weeks. Risedronate increased the bone continuity of fracture at the 7th week, but this phenomenon was not found at the 10th week. Delayed fracture healing occurred locally at the fracture site. At the 7th week, Risedronate may promote cartilage cells proliferating at the fracture site, increase the density of bone trabeculae and the connection of bone trabeculae, thicken the bone cortex showing better fracture healing than OPF‐Saline groups. However, these parameters did not continue during the 7th and 10th weeks. Comparing the first and the later 3 weeks, the rats in group OPF‐RD accelerated the local fracture healing in the first 3 weeks compared to the last 3 weeks, which is consistent for the BMD and BMC among each group. Through evaluation of bone mineral density and bone mineral content, risedronate could dramatically increase the BMD at the fracture site, and effect dramatic reduction of BMC by risedronate at the fracture site. Through load testing, Risedronate can increase the structural strength and mechanical indexes of the new callus.

Of course, this experiment still has deficiencies. In this experiment, due to experimental funding issues, further studies on the use of risedronate sodium in the treatment of osteoporotic fractures leading to delayed fracture healing were not further explored. At present, the problem of experimental funding has been resolved. This deficiency will be our main experimental exploration direction in the next step. Moreover, due to the problem of related testing equipment, in the test of BMD and BMC in rats, only the measurement of fracture healing was performed, and the distal fracture measurement was not performed to further understand the effect of risedronate on systemic BMD and BMC after osteoporotic fracture. At present, we have also actively contacted relevant animal experiment centers. This will not be an issue in the next stage of our experiment. The conclusion of this article is based on the osteoporosis model of ovariectomized rats. Whether it is suitable for other types of osteoporosis animals requires further study. This shortcoming will also be our main exploration direction in the next stage.

### Increasing Local Bone Mass in the Early Stage

*Via* X‐ray and pathologically, our data indicated that risedronate treatment promoted the callus forming at the fracture site during the following 6 weeks after osteoporotic fracture by X‐ray, increased the local bone mineral density, and accelerated the fracture healing during the first 3 weeks, but delayed facture healing in the later 3 weeks. Risedronate increased the bone continuity of fracture at the 7th week, but this phenomenon was not found at the10th week. Delayed fracture healing occurred locally at the fracture site. At the 7th week, Risedronate may promote cartilage cells proliferating at fracture site, increase the dense of bone trabeculae and the connection of bone trabeculae, thicken the bone cortex showing better fracture healing than OPF‐Saline groups. However, these parameters did not continue during the 7th and 10th weeks. Comparing the first and the later 3 weeks, the rats in the OPF‐RD group accelerated the local fracture healing in the first 3 weeks but not in the last 3 weeks. Ito *et al*.^31^ also found that BP drugs exert their effects mainly through the increasing local bone mass, which would improve the mechanical properties of local bone tissue microstructure, and through an increase in bone trabecular connectivity.

### Damage of the Bone Remodeling for Long Term Use

However, at the 10th week, risedronate administration obviously delayed fracture healing in group OPF‐RD rats. Therefore, the question arises as to whether the long‐term use of risedronate impairs the ability of bone remodeling and leads to delayed healing of atypical fractures? A dynamic variation phenomenon was found in the measurement of BMD and BMC at the fracture site in which BMD increased and BMC decreased in a time‐dependent manner, which may explain the cumulative effects of risedronate at the local fracture. It is known, in fact, that BP drugs possess a more than 10 years half‐live in bones^32^, which has been confirmed by a group of data showing that pamidronate could still be detected in the urine of patients 8 years after stopping taking BP drug^33^. Therefore, it is plausible that BP drugs accumulating locally in the fracture would damage the ability of bone remodeling and limit the third stage of fracture healing which is the formation process from braided bone to plate bone. In all, our study supports the notion that long‐term use of BP drugs for prevention and treatment of osteoporosis will damage the ability of bone remodeling, leading to the formation of new fractures, that is, atypical fractures.

Our results demonstrate that short‐term administration of residronate increased the BMD at the fracture site as well as their biomechanical characteristics of the callus indicated by the maximum load and maximum load recovery rate. This study also suggests that after the intramedullary nail fixation and the use of risedronate sodium, both fretting and risedronate sodium at the broken end of the fracture can promote the mineralization of the callus, resulting in the transformation of the larger fibrous callus initially formed at the fracture end to the osseous callus. At the early stage of bone remodeling, the volume of the callus became larger and the mechanical properties improved continuously. Moreover, a certain degree of mechanical stimulation promoted bone marrow mesenchymal stem cells migrating into osteoblasts. After the formation of mineralized callus, the new callus entered the remodeling stage. At this stage, the change of mechanical stress mainly affects the formation of bone. Observing the rat tibia fracture model, Sigurdsen *et al*.^34^ found that axial compression could promote the increase of mineralization degree, mechanical strength and stiffness of bone compared with axial tension.

### Research Prospect and Possible Scheme

At present, few scholars have further explored further studies on the use of risedronate sodium in the treatment of osteoporotic fractures that lead to delayed fracture healing. However, there is still a lack of relevant experimental studies on further understanding the effects of risedronate on the body BMD and BMC after osteoporotic fractures. The authors believe that research in these areas will be a research hotspot to explore the effect of risedronate on the healing process of osteoporotic fractures. This experimental group is very interested in exploring the correlation between risedronate sodium and osteoporotic fractures. We will continue the next phase of research based on this experimental study. In the next stage of experimental research, we will focus on exploring the correlation between the dosage of phosphate drugs, the frequency of drug use and the time of drug use for osteoporotic fractures, which will provide the basis for clinical dosing.
